# Evaluation of Available Energy and Standardized Ileal Digestibility of Amino Acids in Fermented Flaxseed Meal for Growing Pigs

**DOI:** 10.3390/ani14020228

**Published:** 2024-01-11

**Authors:** Zixi Wei, Lei Xu, Yao Guo, Baozhu Guo, Chunxiang Lu, Wenjuan Sun, Yanpin Li, Xianren Jiang, Xilong Li, Yu Pi

**Affiliations:** 1 Key Laboratory of Feed Biotechnology of Ministry of Agriculture and Rural Affairs, Institute of Feed Research, Chinese Academy of Agricultural Sciences, Beijing 100081, China; 13126830289@163.com (Z.W.); xlei0611@163.com (L.X.); yg410@scarletmail.rutgers.edu (Y.G.); sunwenjuan@caas.cn (W.S.); liyanpin@caas.cn (Y.L.); jiangxianren@caas.cn (X.J.); 2 Precision Livestock and Nutrition Unit, TERRA Teaching and Research Centre, Gembloux Agro-Bio Tech, University of Liege, 5030 Gembloux, Belgium; 3 Zhangjiakou Animal Husbandry Technology Extension Station, Zhangjiakou 075000, China; guobaozhu957@163.com (B.G.); 18713512402@163.com (C.L.)

**Keywords:** fermented flaxseed meal, digestion and metabolism, digestible energy, metabolic energy, standardized ileal digestibility of amino acids, growing pig

## Abstract

**Simple Summary:**

The cyanogenic glycosides (CGs) in flaxseed meal (FSM) severely limit its application in pig diets. Microbial fermentation is an effective method to reduce CGs in FSM. However, currently, there is limited information on the nutritional value of fermented flaxseed meal (FFSM) as a protein source in pig diets. In this study, we significantly reduced the CG content and enhanced the nutrient profile of FSM through microbial fermentation. Subsequently, we conducted a thorough evaluation of the nutritional value of FFSM, providing valuable information on the available energy value and amino acid standard ileal digestibility of FFSM. These data are crucial for the application of FSM in pig feed formulations, contributing to the development of more scientific and systematic feeding strategies to enhance pig production.

**Abstract:**

Flaxseed meal (FSM) is a byproduct of flaxseed oil extraction which has rich nutritional value and can be used as a high-quality new protein ingredient. However, the anti-nutrient factor (ANF) in FSM restricts its potential application in feed. The strategy of microbial fermentation is a highly effective approach to reducing ANF in FSM and enhancing its feeding value. However, evaluation of the nutritional value of fermented flaxseed meal (FFSM) in growing pigs has not yet been conducted. Thus, the purpose of this study was to investigate the nutritional value of FFSM in growing pigs and comparison of the effect of fermentation treatment on improving the nutritional value of FSM. Two experiments were conducted to determine the available energy value, apparent digestibility of nutrients, and standard ileal digestibility of amino acids of FSM and FFSM in growing pigs. The results showed as follows: (1) Fermentation treatment increased the levels of crude protein (CP), Ca and P in FSM by 2.86%, 9.54% and 4.56%, while decreasing the concentration of neutral detergent fiber (NDF) and acid detergent fiber (ADF) by 34.09% and 12.71%, respectively (*p* < 0.05); The degradation rate of CGs in FSM was 54.09% (*p* < 0.05); (2) The digestible energy (DE) and metabolic energy (ME) of FSM and FFSM were 14.54 MJ/kg, 16.68 MJ/kg and 12.85 MJ/kg, 15.24 MJ/kg, respectively; (3) Compared with FSM, dietary FFSM supplementation significantly increased the apparent digestibility of CP, NDF, ADF, Ca, and P of growing pigs (*p* < 0.05) and significantly increased the standard ileal digestibility of methionine (*p* < 0.05). These results indicate that fermentation treatment could effectively enhance the nutritional value of FSM and provide basic theoretical data for the application of FFSM in pig production.

## 1. Introduction

Soybean meal (SBM), recognized as the primary protein supply in animal feed, is grappling with issues related to resource scarcity and rising prices. This scenario has encouraged the livestock industry to look for alternative protein feed resources to solve the SBM supply problem. An underutilized byproduct from flaxseed oil extraction, flaxseed meal (FSM), is valued for its robust nutritional content, which includes rich protein levels and unsaturated fatty acids. This quality positions FSM as a potential innovative protein feed alternative to replace SBM [1]. However, a critical concern with FSM is its anti-nutritional factors (ANFs), notably cyanogenic glycosides (CGs). When metabolized inside the animal’s body, CGs can theoretically induce cellular asphyxiation due to hydrogen cyanide (HCN) production. Previous research indicates that overreliance on FSM in livestock and poultry diets can impair nutrient digestion and absorption and reduce animal feed intake, consequently negatively affecting growth performance [2,3].

Extensive research suggests microbial fermentation as an optimal method for degrading ANFs and enhancing nutritional value [4,5,6]. Evidence reveals that the co-fermentation of corn-soybean meal feed with *Bacillus subtilis* and *Enterococcus faecium* notably mitigates the levels of ANFs while simultaneously boosting the soluble protein content [7]. Furthermore, a study utilizing the β-glucosidase M-2 strain isolated from bovine feces for FSM fermentation achieved an impressive 89% clearance of CGs and increased CP content. Importantly, poultry research has shown that *Aspergillus niger* and *Candida utilis* fermentation of FSM not only reduces HCN levels but also enriches the nutritional composition of FSM, thus augmenting FSM utilization in poultry diets [8]. Our research aim, in light of these facts, was to use fermentation techniques to decrease CG prevalence and primary ANF in FSM, enhancing its nutritional value and resulting in escalated usage in pig farming. It is noteworthy that, despite some studies exploring FSM’s nutritional value in pig farming [9,10], there remains a noticeable void in research on the nutritional value assessment and use of solid-state fermented FSM (FFSM) in growing pigs.

To fully assess FFSM’s potential in swine production, a tiered approach is required to obtain detailed nutritional information and conduct a comprehensive evaluation. Hence, this study strives to form a robust nutritional value database for FFSM and investigate the effect of fermentation treatment on the nutritional value enhancement of FSM. Based on this objective, our research includes conducting a thorough chemical analysis of FSM’s nutritional components pre- and post-fermentation and evaluating the digestive energy (DE), metabolizable energy (ME), gross energy (GE), apparent total tract digestibility (ATTD), and standardized ileal digestibility (SID) of amino acid in growing pigs fed with FFSM.

## 2. Materials and Methods

The animal procedures in this study were approved by the Institute Animal Care and Use Committee of the Institute of Feed Research of the Chinese Academy of Agricultural Sciences. The trial was conducted at the Tianpeng experimental farm, located in Lang fang, Hebei province (IFR-CAAS20221025).

### 2.1. FSM Fermentation

Flaxseed meal (FSM) was purchased from Hebei Kaikuo Food Group Co., Ltd. (Zhangjiakou, China). The procedure for fermentation sample preparation commenced by finely grinding the FSM, which was then passed through a 40-mesh screen. The next step involved creating a mixture in a 1:0.6 ratio of FSM to water, with molasses constituting 2% of this concoction. This mixture was then inoculated with a 4% solution of *Bacillus subtilis* (with a density of 1.8 × 10^8^ CFU/mL). After thorough mixing, the resultant formula was packed into fermentation bags, sealed, and stored for 14 days at 37 °C. The strain *Bacillus subtilis* used in this study was isolated by the Pig Nutrition and Feed Innovation Team of the Feed Research Institute of the Chinese Academy of Agricultural Sciences. The strain was identified as *Bacillus subtilis* FRI and maintained in the China General Microbiological Culture Collection Center (CGMCC No. 28734). It has a high ability to degrade CGs in FSM.

### 2.2. Animals and Experimental Designs

Exp. 1: We designated 18 crossbred growing pigs, averaging a body weight (BW) of 53.64 ± 5.04 kg, into three dietary treatment groups. Each treatment group encompassed six replicated units. A conventional corn–soybean meal served as the baseline diet for the control group, while the two experimental groups had 30% of the corn–soybean meal in the basal diet substituted with either FSM or FFSM (as outlined in Table 1). To ascertain the digestible energy (DE) and metabolizable energy (ME) content within the FSM and FFSM, we employed both the total collection method for feces and urine and the comparative difference method.

During the experimental phase, all the pigs were individually housed in stainless-steel pens and underwent a 7-day acclimation period. Throughout this time, we incrementally replaced their regular feed with the assigned experimental diet until a complete switch was achieved by the end of the week. The pigs were maintained in an environment held steadily at 22 ± 2 °C and provided continuous access to drinking water. The daily food allocation was set at 4% of the BW, evenly split and administered at 8:00 and 16:00 daily, with records of daily feed consumption. After feeding, routine cleaning of the enclosures was conducted. The full duration of the experiment spanned 10 days; 7 days were dedicated to diet acclimatization, with the succeeding 3 days dedicated to the systematic collection of feces and urine.

Exp. 2: In a separate trial, six crossbred growing pigs, with an average weight of 50.5 ± 6.64 kg and fitted with T-cannulas at the terminal ileum according to the method [11], were distributed into a 3 × 3 Latin square design encompassing three distinct periods and three diet treatments; each treatment contained two replicates. The dietary treatments entailed a nitrogen-free (N-free) diet and two test diets comprised of FSM and FFSM (Table 2). The N-free diet—which was utilized to measure basal ileal endogenous nitrogen losses—included 73.00% corn starch and 15.00% sucrose, while the experimental diets were enriched with 40% FSM as the exclusive dietary nitrogen source. A 0.3% chromium trioxide fortification was made to all the diets to serve as an indigestible marker. We conducted evaluations of the apparent and SID of amino acids in FSM and FFSM, employing both the indicator method and the direct method to obtain these measurements.

### 2.3. Sample Collection

Exp. 1: Throughout the sample collection phase of the experiment, we meticulously gathered and dried the daily feed residues to record accurate intake data. For each pig, fecal output over a 24 h interval was collected in a metabolic crate. Immediately after collection, the fecal matter was carefully bagged, distinctly labeled, and then promptly placed in a freezer at −20 °C to stop fermentation.

In tandem with the fecal collection, we also gathered urine using the complete collection method. To this end, 50 mL of 6N hydrochloric acid (HCl) was preemptively added to the collection trays located beneath the metabolic crates [12]. All 24 h urine output from the test subjects was precisely measured and recorded. Following thorough mixing of the day’s urinary output, a 1/20 aliquot was then frozen at −20 °C. Upon the experiment’s conclusion, we thawed the pig’s fecal and urine samples, amalgamated them for each corresponding period, and then proceeded to sample for chemical analysis. These fecal subsamples were then dried at a consistent 65 °C for 72 h in a forced-air oven. The urine samples, each 5 mL in volume, were dried at the same temperature, using a quantitative filter paper in a crucible, for 8 h [13].

Exp. 2: The use of stainless-steel metabolic crates, the controlled environmental conditions, and the feeding methodologies were all maintained consistently with those in Exp. 1. After a 14-day recovery period, we initiated the trial. This experiment was segmented into three 7-day phases, with the 5 days gauged for dietary adaptation and the final 2 days allocated for digesta collection. Adhering to the protocol delineated in our referenced methodology [14], we affixed a Ziplock bag to the barrel of the cannula on days 6 and 7 of each trial period, allowing the collection of ileal digesta samples from 8:00 to 17:00 over 9 h. We vigilantly replaced the bags at minimum intervals of 30 min as they filled with digesta, and the contents were then immediately stored at −20 °C. Following the trial’s end, the accumulated digesta were defrosted, pooled per individual animal and testing period, uniformly mixed, and then segmented into samples ready to undergo lyophilization utilizing a vacuum freeze-dryer from Tofflon Freezing Drying Systems, located in Shanghai’s Minhang District.

### 2.4. Chemical Analysis

Following the methodologies outlined in the referenced literature [15], we conducted analyses of dry matter (DM; method 930.15), crude protein (CP; method 990.03), crude fat (EE; method 920.39), calcium (Ca; method 967.30), phosphorus (P; method 965.17), and crude ash (Ash; method 942.05) in the FSM, FFSM, N-free diet, and fecal samples. Total N content was calculated as CP/6.25. We quantified neutral detergent fiber (NDF) and acid detergent fiber (ADF) using the procedure specified in the relevant research [16]. The energy content of the fecal and urine samples was assessed using an automatic oxygen and nitrogen calorimeter. We determined the analytical amino acid and chromium levels following the recommended methods [17,18]. The content of the CGs was ascertained through the colorimetric method, adhering to the People’s Republic of China National Standard GB/T13084-2006 [19].

### 2.5. Calculations

In Exp. 1, the nutrient digestibility, the apparent total tract digestibility of GE, and energy values (DE and ME) of the diets and raw materials were calculated with reference to the equations [20], as follows:

DE_d_ = (GE_i_ − GE_f_)/F_i_

DE_f_ = [DE_d_ − (100% − X%) × DE_d_]/X%

ME_d_ = (GE_i_ − GE_f_ − GE_u_)/F_i_

ME_f_ = [ME_d_ − (100% − X%) × ME_d_]/X%

ATTD = (GE_i_ − GE_f_)/GE_i_,

where DE_d_, ME_d_ are the digestible and metabolic energy values in the diet (MJ /kg); DE_f_, ME_f_ are the digestible and metabolic energy values in the fecal sample (MJ /kg); Fi is the total feed intake per pig; GE_i_ is the total energy intake per pig (the product of the GE value in the diet and Fi); GE_f_ is the total GE content in the feces per pig (the product of the fecal GE content and the total fecal sample weight); GE_u_ is the total GE content in the urine per pig (the product of the urine GE content and the total urine volume); and X% is the proportion of the energy-supplying portion of the base diet replaced by the raw material to be measured.

In Exp. 2, the apparent ileal digestibility (AID) and SID of AA were calculated according to method [21].

AID (%) = [1 − (AA_digesta_/AA_diet_) × (Cr_diet_/Cr_digesta_)] × 100,

in which AA_digesta_ and Cr_digesta_ represent the concentrations of AA and Cr in ileal digesta (g/kg DM). AA_diet_ and Cr_diet_ represent the concentration of AA and Cr in the diet (g/kg DM).

Ileal endogenous loss of AA (IAA) was calculated according to the following formula:

IAA (g/kg DM) = (AA_digesta_) × (Cr_diet_/Cr_digesta_),

in which AA_digesta_ and Cr_digesta_ represent the concentrations of AA and Cr (g/kg DM) in ileal digesta of an N-free diet. Cr_diet_ represents the concentration of AA and Cr (g/kg DM) in the N-free diet (g/kg DM). 

The SID was calculated using the following equation:

SID (%) = AID + (IAA/AA_diet_) × 100

### 2.6. Statistical Analysis

The data in Experiment 1 were analyzed using a completely randomized design with repeated measures, followed by one-way ANOVA and Tukey’s multiple comparison test in SPSS 20.0 software (SPSS Inc., Chicago, IL, USA). The data in Experiment 2 were analyzed using the PROC MIXED procedure in SAS (SAS Inst. Inc., Cary, NC, USA), with pigs serving as experimental units. In the statistical model, diets were considered fixed effects, while pigs and period were treated as random effects. The treatment means were calculated using the LSMEANS statement in SAS. Analysis of variance was conducted employing Tukey’s multiple range test to determine significant differences among the groups. Statistical significance was determined at *p* < 0.05 and tendencies at 0.05 ≤ *p* < 0.10.

## 3. Results

### 3.1. Effects of Fermentation on the Chemical Composition of FSM and FFSM

The chemical compositions of FSM and FFSM used in Exp. 1 and Exp. 2 are detailed in Table 3. The fermentation of the FSM led to a rise in the content of the CP, Ca, and P by 2.86%, 9.54%, and 4.56%, respectively, but these increases did not show statistically significant differences. However, the NDF and ADF saw a significant decrease by 34.09% and 12.71%, respectively (*p* < 0.05). Additionally, the fermentation process led to a notable reduction in the CGs content in the FSM, dropping from 184.94 mg/kg to 84.91 mg/kg, signifying a degradation rate of 54.09% (*p* < 0.05).

### 3.2. Apparent Digestibility of Chemical Components in Exp. 1

As articulated in Table 4 and Table 5, the apparent digestibility of the main chemical components in FSM diets and FFSM diets had no significant difference in Table 4, but the digestibility of CP, ADF, NDF, Ca, P, and GE was significantly higher in the FFSM group compared to the FSM group in Table 5 (*p* < 0.05), and there was a trend (*p* = 0.086) for an improvement in the apparent digestibility of OM. 

### 3.3. Energy Value and Nitrogen Balance in Exp. 1

As shown in Table 4 and Table 5, the DE and ME were higher in the FFSM group compared to the FSM group (*p* < 0.01), with the DE values of FSM and FFSM at 14.54 MJ/kg and 16.68 MJ/kg respectively. The ME values for FSM and FFSM were 12.85 MJ/kg and 15.24 MJ/kg, respectively. In terms of the nitrogen balance in diets, the nitrogen intake, fecal nitrogen, urine nitrogen, and nitrogen retention were higher in FSM and FFSM compared to the basic diet (*p* < 0.01), though there was no significant difference in nitrogen balance between FSM and FFSM.

### 3.4. Ileal Digestibility of CP and AA in Exp. 2

Comparing the FSM group, feeding FFSM significantly amplified the AID of methionine (*p* < 0.05), and the SID of methionine was more elevated in FFSM than in FSM (*p* < 0.05). However, there were no significant differences in the AID and SID of other AAs (*p* > 0.05) as presented in Table 6 and Table 7.

## 4. Discussion

Flaxseed meal (FSM) has wide-ranging applications in the animal feed industry and can potentially serve as a high-quality protein ingredient for livestock and poultry. However, high levels of FSM in the diet can lead to adverse effects due to the presence of CGs, which has significantly hampered the broader use of FSM in animal feed. Studies have demonstrated the significant potential of microbial fermentation in enhancing the nutritional values of feed ingredients. Previous research has shown that fermenting SBM with yeast and *Bacillus* spores results in a substantial increase in CP, lysine, methionine, and total AA content [22]. Similarly, another study revealed that fermenting peanut meal using *Bacillus velezensis* and *Pediococcus acidilactici* increases the levels of CP and AA while reducing crude fiber, phytic acid (PA), and aflatoxin B content. Additionally, it has been reported that *Bacillus* spores display substrate specificity in the microbial degradation of cyanides [23], supporting the potential reduction in feed ANFs by fermentation. Here, we employed *Bacillus subtilis* for the fermentation of FSM. The result showed a substantial reduction in CG content from 184.9 to 84.9 mg/kg, achieving a degradation rate of 54.1%. Furthermore, we observed improvements in several key nutritional parameters in the FFSM. The FFSM displayed increased the levels of CP, Ca, and P. Besides methionine, the levels of other amino acids also increased post-fermentation. Moreover, fiber degradation improved, corroborating previous research findings [24,25]. The enhancement of nutritional components in FFSM is potentially due to the role of microorganisms during the metabolic process. The microbes appear to efficaciously utilize the ANFs and fibers in the FSM, metabolizing them into smaller molecules that can be more easily digested and absorbed by the body, thereby boosting FSM’s overall nutritional value. Interestingly, our study found lower levels of CP and CGs compared to previous studies, potentially due to several factors, such as divergent flaxseed varieties and variations in fermentation environments [26,27].

In the pursuit of precision feeding and the application of FFSM into swine diets, it is essential to acquire accurate nutritional parameters and comprehend the shifts in nutrient digestibility. At present, there is a noted paucity of data on the nutritional value of both FSM and FFSM as feed ingredients. Our study aimed to bridge this research gap by evaluating the energy values and nutrient digestibility of FSM and FFSM. Our findings highlighted that the DE of FSM was 14.54 MJ/kg, while that for FFSM was 16.68 MJ/kg. Additionally, the ME of FSM was 12.85 MJ/kg, and the FFSM exhibited an ME of 15.24 MJ/kg. Compared to previous studies [28,29], our results appear to be slightly higher in the value of available energy in FSM, a discrepancy that might be attributed to the different processing methods. These methods influence the residual oil content in FSM, consequently affecting the variations in ether extract (EE) content [29,30]. It is particularly noteworthy that in our study, the EE content in FSM was higher than that of the National Research Council (NRC), suggesting that the FSM used in our investigation was rich in fat, owing to a greater residual oil content. This factor is likely linked to the elevated energy values we observed.

Prior research has comprehensively explored the factors influencing the energy values in pig diets, which include the impact of fiber content on the values of DE and ME. These studies have consistently demonstrated a negative correlation between fiber content (particularly NDF) and the levels of DE and ME. This implies that reducing the fiber content can enhance the energy value of feed [31,32]. In our study, we found that the levels of NDF and ADF in FFSM decreased by 34.09% and 12.71%, respectively, as compared to FSM. The ATTD of NDF and ADF in FFSM was significantly higher than in FSM. Furthermore, during the fermentation process, we added 2% molasses as an energy substrate for the growth of *Bacillus subtilis*. This addition of molasses is expected to increase the energy value in FFSM. Therefore, the combination of diminished NDF and ADF content and the addition of molasses are believed to be the main factors driving the observed increase in energy value (DE and ME) of FFSM. The National Research Council (NRC, 2012) did not provide sufficient data on the energy value and nutrient digestibility of FFSM. The energy value data for FFSM presented in this study serves as a vital reference for future pig feed formulations, facilitating the development of more scientifically and systematically designed pig feeding strategies to enhance production practices.

Flaxseed meal contains significant amounts of Ca and P. However, a majority of these minerals are bound with PA, forming phytate complexes, which are challenging for pigs to utilize [33]. Conversely, our findings indicate that the ATTD of Ca and P in the FFSM group is significantly higher than that in the FSM group. This suggests that the fermentation process aids in the hydrolysis of phytate-bound Ca and P, rendering them into more bioavailable and effective forms. Furthermore, the rise in fecal nitrogen, urinary nitrogen, and nitrogen retention values was observed to be positively correlated with the increased nitrogen intake post-fermentation. This indicates that the fermentation of FSM may enhance nitrogen retention by promoting feed intake.

Accurate assessment of amino acid digestibility in FFSM is essential for its effective utilization in pig feed formulations. Multiple studies have demonstrated that microbial fermentation of protein ingredients can break down large protein molecules into free peptides, thus enhancing amino acid utilization [34,35]. However, specific effects can vary depending on the chosen microbial strains. The results of this study align with previous research, indicating that fermentation significantly improves the AID and SID of methionine. Furthermore, this study employed solid-state fermentation with *Bacillus subtilis*, which significantly reduced the content of the CGs and fiber (NDF and ADF). This helps to alleviate the adverse impact of ANFs and fiber on amino acid digestibility. In addition, in this study the SID of AA in FSM was observed predominantly higher when compared to the results from Eastwood’s research [28]. The variations in pig body weight, feed source and analysis method, diet composition, and CP content may account for the observed differences in SID.

## 5. Conclusions

In summary, microbial fermentation has been found to enhance the nutritional value of FSM by increasing the DE and ME, as well as the SID of methionine. According to this experiment, the DE and ME values for FFSM were measured to be 16.68 MJ/kg and 15.24 MJ/kg, respectively. These findings provide valuable data support for the utilization of FFSM in pig production.

## Figures and Tables

**Table 1:** Ingredient composition and chemical components of the diets in Exp. 1 (as-fed basis).

Items	Basal Diet	FSM Diet	FFSM Diet
Ingredients (%)			
Corn	79.80	55.86	55.86
SBM, CP 46%	17.02	11.91	11.91
FSM	-	29.05	
FFSM	-		29.05
Dicalcium phosphate	1.36	1.36	1.36
Salt	0.30	0.30	0.30
Limestone	1.02	1.02	1.02
Premix ^1^	0.50	0.50	0.50
Total	100.00	100.00	100.00
Analyzed chemical components levels (%)			
GE, MJ/kg	15.51	16.47	17.70
DM	86.35	87.90	84.55
CP	13.59	17.21	18.05
NDF	9.48	14.43	13.15
ADF	3.61	7.26	6.82
EE	2.04	3.00	2.17
Ash	4.77	6.59	5.48
Ca	0.65	0.72	0.81
P	0.65	0.87	1.04
Essential amino acids			
Arginine	0.86	1.26	1.35
Histidine	0.41	0.50	0.51
Isoleucine	0.56	0.69	0.70
Leucine	1.38	1.58	1.64
Lysine	0.73	0.91	1.02
Methionine	0.25	0.34	0.39
Phenylalanine	0.67	0.87	0.89
Threonine	0.56	0.75	0.75
Tryptophan	0.14	0.27	0.30
Valine	0.73	0.92	0.92
Non-essential amino acids	0.00	0.00	0.00
Alanine	0.82	0.97	0.98
Asparagine	1.34	1.78	1.78
Cystine	0.27	0.36	0.37
Glutamine	2.39	3.61	3.62
Glycine	0.57	0.85	0.88
Serine	0.70	0.91	0.97
Tyrosine	0.70	0.54	0.55

Notes: SBM = soybean meal; FSM = flaxseed meal; FFSM = fermented flaxseed meal; GE = gross energy; DM = dry matter; CP = crude protein; EE = crude fat; NDF = neutral detergent fiber; ADF = acid detergent fiber; P = phosphorus; Ca = calcium. ^1^ Premix supplied per kg of diet: vitamin A, 35.2 mg; vitamin D3, 7.68 mg; vitamin E, 128 mg; vitamin K3, 8.16 mg; vitamin B1, 4 mg; vitamin B2, 12 mg; vitamin B6, 8.32 mg; vitamin B12, 4.8 mg; Niacin, 38.4 mg; Calcium pantothenate, 25 mg; Folic acid, 1.68 mg; Biotin, 0.16 mg; Zn (ZnSO_4_·H_2_O), 110 mg; Copper (CuSO_4_·5H_2_O), 125 mg; Iron (FeSO_4_·H_2_O), 171 mg; Cobalt (CoCl_2_), 0.19 mg; Manganese (MnSO_4_·H_2_O), 42.31 mg; Iodine (Ca(IO_3_)_2_), 0.54 mg; Selenium (Na_2_SeO_3_), 0.19 mg.

**Table 2:** Ingredient composition and chemical components of the diets in Exp. 2 (as-fed basis).

Items	FSM Diet	FFSM Diet	N-Free Diet
Ingredients (%)			
Corn starch	37.29	37.29	73.00
FSM	40.00	-	-
FFSM	-	40.00	-
Sucrose	20.00	20.00	15.00
Cellulose acetic acid	-	-	4.00
Soybean oil	-	-	3.00
Dicalcium phosphate	0.60	0.60	2.32
Potassium carbonate	-	-	0.30
Magnesium oxide	-	-	0.10
Limestone	1.06	1.06	1.03
Salt	0.45	0.45	0.45
Chromic oxide	0.30	0.30	0.30
Premix ^1^	0.30	0.30	0.50
Analyzed chemical components levels (%)			
GE, MJ/kg	16.08	16.06	14.87
DM	92.24	91.66	91.18
CP	10.46	11.10	0.41
NDF	8.22	7.63	1.13
ADF	4.89	4.80	0.18
Essential amino acids			
Arginine	0.88	0.90	0.02
Histidine	0.17	0.23	0.01
Isoleucine	0.41	0.44	0.01
Leucine	0.58	0.65	0.03
Lysine	0.35	0.45	0.01
Methionine	0.10	0.18	0.01
Phenylalanine	0.45	0.47	0.02
Threonine	0.44	0.45	0.01
Tryptophan	0.15	0.18	-
Valine	0.51	0.43	0.32
Non-essential amino acids			
Alanine	0.48	0.55	0.02
Asparagine	1.14	1.13	0.03
Cystine	0.15	0.11	0.01
Glutamine	1.89	2.65	0.07
Glycine	0.66	0.70	0.03
Serine	0.53	0.60	0.02
Tyrosine	0.25	0.23	0.01

Notes: FSM = flaxseed meal; FFSM = fermented flaxseed meal; GE = gross energy; DM = dry matter; CP = crude protein; NDF = neutral detergent fiber; ADF = acid detergent fiber. ^1^ The vitamin and trace element premix compositions were the same as those in Exp. 1.

**Table 3:** Effect of fermentation on chemical components of FSM (%, as-DM basis).

Items	FSM	FFSM	SEM	*p*-Value
DM	90.4	85.9	1.93	0.256
CP	29.1	30.0	0.22	0.100
EE	8.9	7.1	0.37	0.087
NDF	32.1 ^a^	21.5 ^b^	0.15	0.001
ADF	16.9 ^a^	15.0 ^b^	0.20	0.026
Ca	0.4	0.4	0.06	0.658
P	0.9	1.0	0.04	0.176
GE (MJ/kg)	19.7 ^b^	20.1 ^a^	0.03	0.024
CGs (mg/kg)	184.9 ^a^	84.9 ^b^	3.09	0.002

Notes: FSM = flaxseed meal; FFSM = fermented flaxseed meal; DM = dry matter; CP = crude protein; EE = crude fat; NDF = neutral detergent fiber; ADF = acid detergent fiber; P = phosphorus; Ca = calcium; GE = gross energy; CGs = cyanogenic glycosides. ^a,b^ Means in the same column with different superscripts exhibit significance difference (*p* < 0.05).

**Table 4:** Effects of feeding diets on available energy, the apparent digestibility of chemical components and nitrogen balance in growing pigs (as-DM basis).

Items	Basal Diet	FSM Diet	FFSM Diet	SEM	*p*-Value
Apparent digestibility (%)					
DM	90.6	86.1	86.5	0.59	0.001
CP	87.0 ^a^	84.4 ^b^	85.9 ab	0.33	0.002
NDF	52.7 ^b^	55.1 ^ab^	58.2 a	0.73	0.002
ADF	48.8	48.2	50.0	0.35	0.106
EE	60.1	64.3	64.5	1.06	0.139
Ash	62.6	61.7	61.2	0.70	0.757
Ca	64.1 ^a^	55.9 ^b^	57.6 b	0.97	0.001
P	66.3 ^a^	60.2 ^b^	63.9 ab	0.85	0.005
OM	91.9 ^a^	87.1 ^b^	88.9 b	0.53	0.001
Carbohydrate	93.7 ^a^	89.9 ^b^	91.2 b	0.46	0.001
GE	89.8 ^a^	85.8 ^c^	87.7 b	0.47	0.001
Energy value (MJ/kg)					
GE	15.5	16.5	17.7	-	-
DE	13.6 ^c^	13.9 ^b^	14.6 a	0.11	0.001
ME	12.6 ^b^	12.7 ^b^	13.4 a	0.12	0.001
ME/DE (%)	93.2	92.2	92.4	0.39	0.568
Nitrogen balance (g/day)					
Nitrogen intake	58.4 ^b^	74.6 ^a^	78.3 a	2.31	0.001
Fecal nitrogen	10.0 ^b^	14.1 ^a^	14.8 a	0.61	0.001
Urine nitrogen	11.8 ^b^	17.9 ^a^	19.1 a	0.82	0.001
Nitrogen retention	37.6 ^b^	43.4 ^a^	45.4 a	0.89	0.001

Notes: FSM = flaxseed meal; FFSM = fermented flaxseed meal; DM = dry matter; CP = crude protein; EE = crude fat; NDF = neutral detergent fiber; ADF = acid detergent fiber; P = phosphorus; Ca = calcium; GE = gross energy; DE = digestive energy; ME = metabolizable energy. ^a,b,c^ Means in the same column with different superscripts exhibit significance difference (*p* < 0.05).

**Table 5:** Effects of feeding FSM and FFSM on available energy and apparent digestibility of chemical components in growing pigs (as-DM basis).

Items	FSM	FFSM	SEM	*p*-Value
Apparent digestibility (%)				
DM	75.2	76.4	1.86	0.520
CP	79.4 ^b^	83.8 ^a^	1.14	0.050
NDF	60.5 ^b^	72.0 ^a^	4.39	0.040
ADF	45.2 ^b^	55.7 ^a^	2.18	0.006
EE	67.3	68.7	2.36	0.786
Ash	60.1	58.4	3.04	0.800
Ca	37.6 ^b^	44.7 ^a^	1.79	<0.050
P	48.9 ^b^	60.2 ^a^	2.35	0.007
OM	75.3	81.1	1.68	0.086
Carbohydrate	77.2	82.7	1.90	0.159
GE	76.3	82.6	1.39	0.017
Energy value (MJ/kg)				
DE	14.5 ^b^	16.7 ^a^	0.36	0.001
ME	12.9 ^b^	15.2 ^a^	0.44	0.001
ME/DE (%)	89.9	90.6	1.37	0.800

Notes: FSM = flaxseed meal; FFSM = fermented flaxseed meal; DM = dry matter; CP = crude protein; EE = crude fat; NDF = neutral detergent fiber; ADF = acid detergent fiber; P = phosphorus; Ca = calcium; GE = gross energy; DE = digestive energy; ME = metabolizable energy. ^a,b^ Means in the same column with different superscripts exhibit significance difference (*p* < 0.05).

**Table 6:** The apparent ileal digestibility of CP and AA in FSM and FFSM (%, as-DM basis).

Items	FSM	FFSM	SEM	*p*-Value
CP	67.3	68.4	1.45	0.630
Essential amino acids				
Arginine	87.6	86.1	1.06	0.325
Histidine	71.0	75.6	1.51	0.051
Isoleucine	83.7	84.4	1.12	0.697
Leucine	78.1	80.1	1.35	0.286
Lysine	70.2	72.4	1.72	0.167
Methionine	78.6 ^b^	85.2 ^a^	1.93	0.001
Phenylalanine	83.3	83.7	1.00	0.700
Threonine	58.1	60.8	2.03	0.414
Tryptophan	78.6	79.0	0.84	0.170
Valine	79.2	77.4	1.71	0.511
Non-essential amino acids				
Alanine	72.3	76.5	1.94	0.153
Asparagine	81.8	79.5	1.20	0.185
Cystine	82.0	83.3	0.66	0.340
Glutamine	82.4	80.3	1.16	0.117
Glycine	62.3	63.3	2.28	0.741
Serine	72.6	75.2	1.53	0.163
Tyrosine	74.0	73.2	1.53	0.699

Notes: FSM = flaxseed meal; FFSM = fermented flaxseed meal; CP = crude protein; AA = amino acid. ^a,b^ Means in the same column with different superscripts exhibit significance difference (*p* < 0.05).

**Table 7:** The standardized ileal digestibility of CP and AA in FSM and FFSM (%, as-DM basis).

Items	FSM	FFSM	SEM	*p*-Value
CP	77.0	78.1	1.62	0.733
Essential amino acids				
Arginine	92.8	90.5	1.05	0.204
Histidine	78.1	80.1	1.39	0.159
Isoleucine	87.4	87.9	1.16	0.688
Leucine	84.2	85.5	1.33	0.464
Lysine	77.3	79.7	1.61	0.371
Methionine	84.9 ^b^	90.4 ^a^	1.42	0.034
Phenylalanine	87.9	88.0	1.04	0.963
Threonine	69.6	72.5	2.31	0.402
Tryptophan	81.1	81.0	0.63	0.904
Valine	84.6	82.0	1.67	0.249
Non-essential amino acids				
Alanine	80.9	84.0	1.91	0.265
Aspartic acid	86.2	84.1	1.19	0.246
Cystine	85.4	86.8	1.30	0.569
Glutamine	84.1	85.9	1.27	0.312
Glycine	73.2	74.4	2.40	0.555
Serine	80.6	83.1	1.65	0.267
Tyrosine	80.9	79.9	1.53	0.609

Notes: FSM = flaxseed meal; FFSM = fermented flaxseed meal; CP = crude protein; AA = amino acid. ^a,b^ Means in the same column with different superscripts exhibit significance difference (*p* < 0.05).

## Data Availability

The data presented in this study are available in the article.
